# Comparative Study on the Properties of Smoke Sheet Rubber Produced by Different Solidification Methods

**DOI:** 10.3390/polym18131593

**Published:** 2026-06-26

**Authors:** Linguang Ruan, Lin Yan, Dandan Yao, Bingguo Liu, Shenghui Guo, Jiawang Yin

**Affiliations:** 1 Faculty of Metallurgy and Energy Engineering, Kunming University of Science and Technology, Kunming 650093, China; 2 Key Laboratory of Unconventional Metallurgy, Ministry of Education, Kunming University of Science and Technology, Kunming 650093, China; 3 Yunnan State Farms Group Co., Ltd., Kunming 650233, China; 4 Yunnan Natural Rubber Industry Group Jiangcheng Co., Ltd., Jiangcheng 665900, China

**Keywords:** natural rubber, coagulation method, pineapple juice, non-adhesive components, mechanical properties

## Abstract

To investigate the effects of coagulation methods on the structure and properties of sheet rubber, this study prepared natural rubber using different coagulation systems, including acetic acid, formic acid, biological coagulants, and pineapple juice, and systematically analyzed their non-rubber components, gel content, molecular weight distribution, rheological behavior, and mechanical properties of the vulcanized rubber. The results indicate that the type of coagulant significantly affects the protein, phospholipid, and gel content. Among these, the pineapple juice gel exhibited the lowest residual protein content, suggesting that the proteases, organic acids, and active components it contains promote the degradation and removal of non-rubber components. GPC and rheological results show that pineapple juice gel and bio-gel samples possess a broad molecular weight distribution and exhibit a more pronounced viscoelastic response at high temperatures. After uniform vulcanization, the differences in hardness, tensile strength, and tear resistance among the various samples were minimal, indicating that the vulcanized network determines the final mechanical properties, while the coagulation method primarily regulates microstructure and processing behavior. This study provides a theoretical basis for the application of bio-coagulants in the processing of green shikigai gum.

## 1. Introduction

Natural rubber is an important class of renewable strategic polymer materials [1]. Due to its excellent elasticity [2], tensile strength [3], fatigue resistance [4], and dynamic mechanical properties [5], it is widely used in tires [6], shock-absorbing products [7], sealing materials [8], and the aerospace industry [9,10]. Smoked sheet rubber, a traditional high-quality rubber product prepared from natural latex through processes such as coagulation [11], sheet pressing, and smoke-drying, exhibits comprehensive properties that depend not only on the molecular chain structure of the rubber hydrocarbons [12] but are also closely related to the content and distribution of non-rubber components such as proteins, phospholipids, and ash [13,14].

In particular, coagulation is a critical step in the primary processing of natural rubber that determines particle aggregation, the retention of non-rubber components, the formation of the gel network, and subsequent processing properties [15]. Traditional sheet rubber production typically employs organic acids such as formic acid and acetic acid as coagulants. While this process is well-established and offers high coagulation efficiency, the acid coagulation process [16,17] primarily relies on pH control to induce latex destabilization. It is difficult to effectively regulate the residual levels of non-rubber components such as proteins and phospholipids. Additionally, this method may lead to high acid consumption, significant wastewater loads, and insufficient batch-to-batch product stability [18]. In recent years, bio-coagulants have garnered attention due to their wide availability, environmental friendliness, and potential ability to regulate non-gel components [19,20]. In particular, pineapple juice contains bromelain, organic acids, and various natural bioactive components. These may promote latex coagulation while simultaneously degrading some proteins and altering inter-particle interactions, thereby influencing the microstructure and macroscopic properties of natural rubber [21].

However, there remains a lack of systematic research into the mechanisms linking coagulation methods to the non-gum components, molecular weight distribution, rheological behavior and vulcanized rubber properties of tobacco sheet gum, which limits the application of green bio-coagulation technology in the processing of high-quality tobacco sheet gum. Unlike previous studies that focused solely on the impact of a single coagulant on a specific performance indicator, this paper systematically compares, for the first time, the effects of four coagulation systems—acetic acid, formic acid, biological coagulants and pineapple juice—on the multi-scale structure of sheet rubber. It establishes the correlation mechanism between ‘coagulation method—microstructure—processing/dynamic properties”, revealing the synergistic regulatory role of proteases, organic acids and natural bioactive components in the pineapple juice coagulant. This provides a theoretical basis and technical reference for the green processing of natural rubber and the preparation of high-quality sheet rubber.

## 2. Materials and Methods

### 2.1. Materials and Preparations

The SCR WF (domestically produced whole milk standard rubber, China) and the Indonesian-imported sheet rubber used in this study are both commercially available industrial-grade products. SCR WF is a technically graded rubber processed from fresh latex (with a dry rubber content of approximately 30–35 wt%) and characterized by minimal impurity content and consistent quality, and it complies with the requirements of the GB/T 8081-2018 standard [22]; formic acid (pH: 2.0–2.5), acetic acid (pH: 2.5–3.0) and the biological coagulant (pH neutral) were all commercially available industrial-grade reagents.

**Figure 1:**
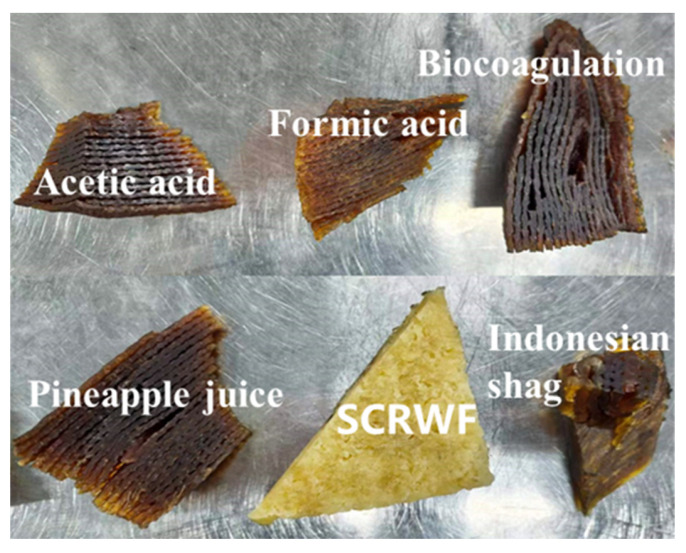
Sample image.

The pineapple coagulant (pH: 3.5–4.0) is a natural pineapple juice obtained by pressing and filtering locally grown fresh pineapples (*Ananas comosus*). This biological coagulant is rich in bromelain and organic acids, which promote latex flocculation whilst breaking down the surface proteins of the latex particles (A sample diagram is shown in Figure 1). The coagulation process is standardized as follows: prepare a coagulant solution at the predetermined concentration ratio; add it to the fresh latex according to the dry rubber mass ratio; after thorough mixing, allow the mixture to stand and mature for 8 h before proceeding to subsequent processing. The coagulation temperature is controlled at 25 ± 2 °C, and the coagulant dosage must be strictly and precisely controlled: when using acetic acid or pineapple juice as the coagulant, the dosage is controlled at 6–10 kg per ton of dry rubber; when formic acid is used as the coagulant, the dosage should be controlled at 5–7 kg per ton of dry rubber; if the small-batch coagulation process is employed, the coagulant must be diluted at least 50-fold before use, and once added, it should be stirred promptly and thoroughly to ensure complete coagulation of the latex.

Repeat tests must be conducted on at least three independent batches for each coagulation system to assess batch-to-batch stability, with the results presented as ‘mean ± standard deviation’.

### 2.2. Test Methods

1. Protein Content Determination

Natural rubber samples were cut into pieces of similar size, and the total nitrogen content was determined using a Kjeldahl nitrogen analyzer (Shandong Drick Instruments Co., Ltd., Model DRK-K616, Jinan, China). The testing procedure followed the GB/T 5009.5—2016 standard [23]. After sample digestion, distillation, and titration, the crude protein content was calculated by multiplying the nitrogen content by the protein conversion factor (6.25). This method is suitable for the quantitative analysis of trace nitrogen-containing components in rubber products.

2. Determination of Phospholipid Content

An inductively coupled plasma optical emission spectrometer (Agilent Technologies, Inc, ICP-OES, Santa Clara, CA, USA) was used to quantitatively analyze phosphorus in the samples. The total phospholipid content was indirectly calculated based on the measured phosphorus content. After sample pretreatment via dry ashing or microwave digestion, the analysis was performed under standard operating conditions (RF power: 1.2 kW; plasma gas flow rate: 15 L/min). This method features a low detection limit and a wide linear range, making it suitable for the accurate analysis of trace phosphorus in rubber matrices.

3. Determination of Gel Content

Accurately weigh approximately 1.0 g of raw rubber sample, place it in a toluene solvent, and allow it to swell and dissolve for 48 h at room temperature (25 ± 2 °C) to fully extract the soluble fraction. Subsequently, perform vacuum filtration through a nylon-66 microporous filter membrane (pore size 0.45 μm) and collect the filtrate. Dry the resulting insoluble gel fraction at 60 °C to constant weight and calculate the gel content (Gel content (%) = Mass of insoluble matter/Mass of original sample × 100). The soluble fraction in the filtrate is further analyzed by gel permeation chromatography (GPC) to determine its molecular weight and distribution. The gel content indirectly reflects the degree of cross-linking and the state of molecular chain aggregation in the rubber.

4. Molecular Weight Determination

Weigh approximately 1.0 mg of the rubber sample and dissolve it in chromatographic-grade tetrahydrofuran (THF). Allow the solution to stand at room temperature for 48 h to ensure complete dissolution of the sample. The solution is then filtered through a nylon-66 filter (0.45 μm), and the filtrate is analyzed using a gel permeation chromatograph (GPC) equipped with a refractive index detector to determine the molecular weight and its distribution (Mw, Mn, PDI). Prior to testing, a standard curve was established using polystyrene standards, with THF as the mobile phase, a flow rate of 1.0 mL/min, and a column temperature of 30 °C. This method is an important tool for characterizing the molecular chain length and distribution of polymers.

5. Tensile Property Testing

Uniaxial tensile tests were conducted on vulcanized rubber specimens using an electronic universal testing machine (Instron, Model Instron-5567, Norwood, MA, USA) at room temperature (23 ± 2 °C). Specimen preparation followed the GB/T 528—2009 standard [24], and dumbbell-shaped specimens were selected.

6. Dynamic rheological testing

A modular, intelligent, advanced rotational rheometer (Anton Paar GmbH, Model MCR302, Graz, Austria) was used to perform temperature-sweep tests on vulcanized rubber samples. The test temperatures were set sequentially to 20, 50, 80, and 110 °C, with a shear strain of 0.5% (within the linear viscoelastic region) and a frequency scan range of 100 to 0.1 Hz.

7. Vulcanization Characteristics Test

Weigh 3–5 g of the rubber compound sample to be tested. Using a rubber processing analyzer (Gaotie Co., Ltd., Model Gaotie-RPA800, Taichung City, Taiwan, China), set the temperature to 143 °C and measure the vulcanization curve under conditions of 1.67 Hz oscillation frequency and 7% strain.

8. Hardness Testing

Hardness testing of vulcanized rubber samples is conducted in accordance with the national standard GB/T 39693.4-2025 [25], using a Shore A hardness tester (Shanghai Chemical Machinery Factory Co., Ltd., Shanghai, China). The sample thickness must be no less than 6 mm; multiple layers may be stacked to meet testing requirements. The indenter of the hardness tester is pressed vertically into the surface of the specimen, and the instantaneous reading is recorded. Measurements are repeated at five different points on each specimen, and the arithmetic mean is taken as the final hardness value. Hardness is an important indicator for evaluating a rubber’s resistance to local indentation and is closely related to the cross-linking density of the material and the dispersion of fillers.

## 3. Results and Discussions

### 3.1. Microstructure

#### 3.1.1. Trace Content

**Figure 2:**
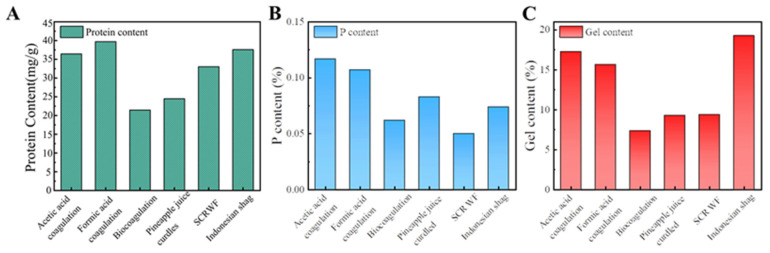
Results of micro-level content analysis: (**A**) Protein (**B**) Phospholipids (**C**) Gel.

As shown in Figure 2, rubber samples treated with different coagulants exhibited significant differences in protein content, phospholipid content, and gel content, and there was an intrinsic correlation among these three parameters [26]. Specifically, regarding protein content (Figure 2A), the ranges for the acetic acid coagulation group, the formic acid coagulation group, and the Indonesian sheet rubber were 35–37.5 mg/g, 37.5–40 mg/g, and 35–37.5 mg/g, respectively, all of which were significantly higher than that of the pineapple juice-cured group (22.5–25 mg/g).

This difference can be attributed to the high concentration of bromelain in pineapple juice, which specifically degrades the protein components in natural rubber latex [27], whereas traditional organic acid coagulants [28] merely induce protein denaturation and flocculation by lowering the pH, but fail to effectively break down the intact structure of protein molecules, resulting in a higher residual protein content in the final rubber product.

Regarding phospholipid content (Figure 2B), the acetic acid and formic acid coagulant groups exhibited higher phospholipid levels, followed by the pineapple juice coagulant group. This trend suggests that the active components in pineapple juice may exert a certain degrading or transforming effect on phospholipids, differing from the mechanism of traditional organic acid coagulants, which simply induce physical aggregation and precipitation of phospholipids by adjusting the pH; specific enzymes or organic acid components potentially present in pineapple juice may participate in the biochemical reactions of phospholipid molecules, partially degrading the phospholipid structure or altering its form, thereby more thoroughly removing phospholipid impurities during the coagulation process.

Furthermore, regarding gel content (Figure 2C), the acetic acid-coagulated group, the formic acid-coagulated group, and the Indonesian tobacco sheet rubber group exhibited significantly higher gel content than the pineapple juice-coagulated group. This phenomenon is primarily attributed to the synergistic effects of non-rubber components (particularly proteins and phospholipids): higher protein and phospholipid content typically leads to the formation of more cross-linking sites, thereby increasing gel content. It is worth noting that although Indonesian tobacco leaf gum has a lower phospholipid content, its gel content remains high, indicating that in addition to proteins and phospholipids, other factors such as the molecular weight distribution of the gum and the rigidity of the molecular chains—characteristics of non-gum components—also have a significant influence on gel formation.

In summary, pineapple juice coagulants, through the synergistic action of their protease and organic acid components, can more efficiently degrade and remove protein and phospholipid impurities from latex, thereby significantly reducing the residual levels of non-latex components and correspondingly affecting the gel network’s ability to form.

#### 3.1.2. Molecular Weight Distribution

**Figure 3:**
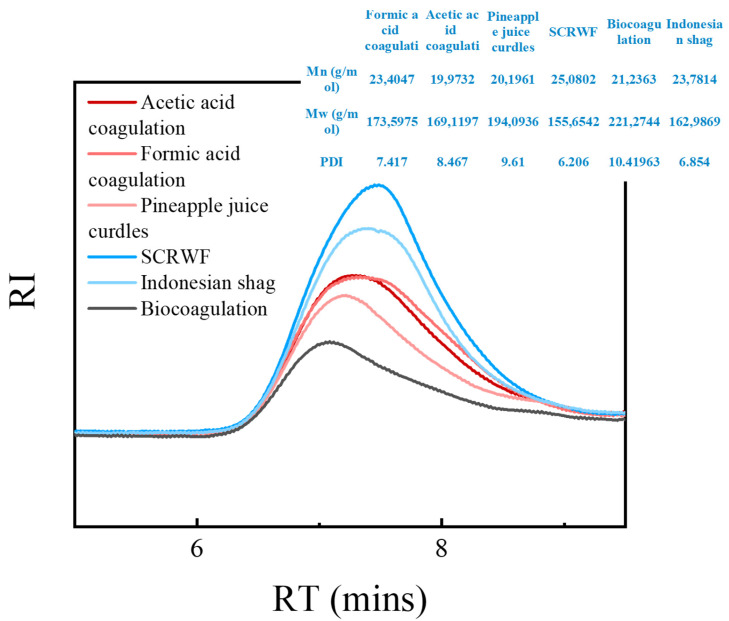
Results of molecular weight distribution analysis.

The gel permeation chromatography (GPC) results shown in Figure 3 and Table 1 [29] indicate that rubber samples prepared using pineapple juice coagulation and bioenzyme coagulation exhibit a broader molecular weight distribution compared to the group treated with conventional organic acid coagulation. This difference is primarily attributed to the lower levels of protein and phospholipid residues in the two novel coagulants. The reduction in non-rubber components weakens their physical binding effect on the rubber molecular chains, making it easier for polymer molecules to form disordered aggregates during coagulation, thereby broadening the molecular weight distribution range; furthermore, the targeted action of specific proteases during the bioenzymatic coagulation process may cause the breakage of some macromolecular chains, while the synergistic effects of various active components in pineapple juice (such as bromelain) may induce selective degradation or rearrangement of rubber molecular chains, further exacerbating the heterogeneity of the molecular weight distribution. These results reveal the significant influence of coagulant type on the microstructure of natural rubber, indicating that regulating the content of non-rubber components can effectively alter the molecular weight distribution characteristics of the rubber.

### 3.2. Performance Testing

**Figure 4:**
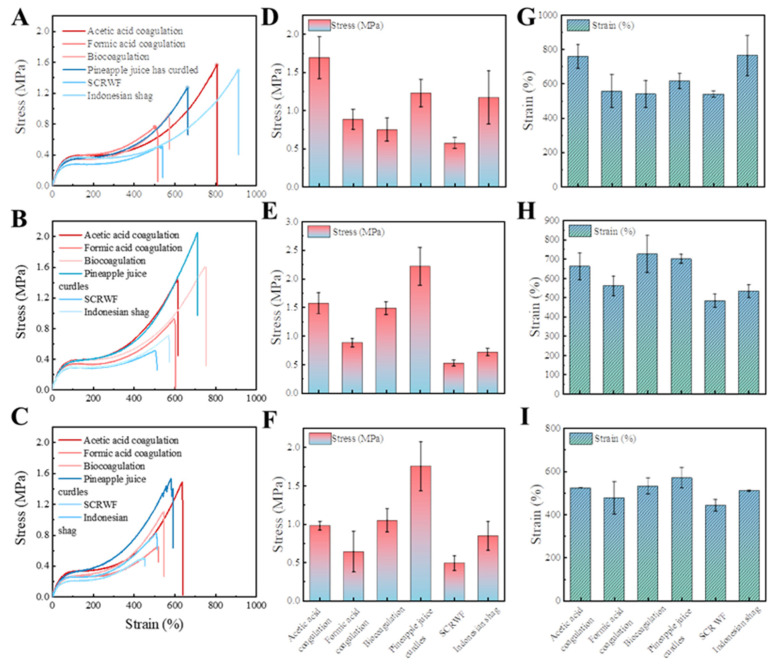
Test results for the mechanical properties of raw rubber in three groups: (**A**–**C**): stress–strain curves for raw rubber under different curing methods; (**D**–**F**): comparisons of stress in raw rubber under different curing methods; (**G**–**I**): comparisons of strain in raw rubber under different curing methods.

Figure 4 shows that the rubber samples prepared using different coagulants exhibited poor batch-to-batch consistency. This was particularly evident in the Indonesian smoke rubber group, where the mechanical properties of all batches except the first one showed a significant decline. This suggests considerable variability in the raw materials or manufacturing processes, which may be closely related to factors such as unstable sources of imported smoke sheet rubber, varying storage conditions, and inconsistent processing histories; in stark contrast, pineapple juice-cured rubber exhibited excellent reproducibility and stable raw rubber properties throughout the entire experimental process. Its stress–strain curve showed a lower initial crystallization strain, indicating reduced molecular chain order.

The rubber molecules were in a more loosely arranged amorphous state, thereby delaying the onset of induced crystallization. At the same time, the tensile modulus increased significantly. This phenomenon can be attributed to the low content of residual proteins and phospholipids, which weaken intermolecular non-covalent interactions and reduce the density of physical cross-linking sites, thereby decreasing the constraints on segmental motion. Meanwhile, the active components in pineapple juice—such as proteases, various organic acids, and natural polysaccharides—may enhance network rigidity through a physical filling effect, forming a functional dispersed phase similar to nanofillers and thereby improving the overall load-bearing capacity of the composite structure [30]; given that the continuous deterioration of Indonesian gum properties and its high inter-batch variability have severely compromised the reproducibility and comparability of experimental results, subsequent research will focus on the top five coagulation systems exhibiting stable performance (acetic acid, formic acid, bio-enzymes, pineapple juice, and SCRWF coagulated gum) to thoroughly investigate the mechanisms by which different coagulants influence the regulation of non-rubber components and the relationship between the final rubber structure and its properties.

**Figure 5:**
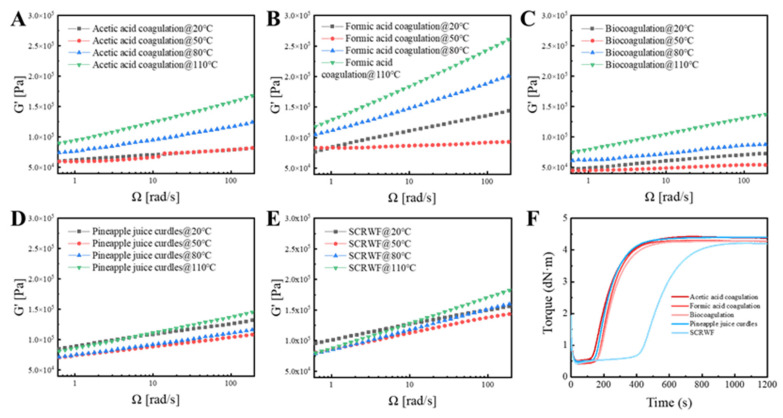
Results of rheological property tests: (**A**) acetic acid coagulation; (**B**) formic acid coagulation; (**C**) enzymatic coagulation; (**D**) pineapple juice coagulation; (**E**) SCRWF; (**F**) results of vulcanization property tests.

The results of the dynamic rheological testing shown in Figure 5 [31] clearly illustrate the temperature-dependent behavior of the storage modulus (G′) for rubber samples prepared using different coagulants; all samples exhibit a typical non-linear trend of ‘first decreasing, then increasing’. In the low-temperature region (<20 °C), rubber molecular chains are restricted by thermal motion and exist in a glassy or highly elastic state; at this point, the storage modulus remains at a high level (typically >10^7^ Pa). As the temperature rises, the mobility of molecular segments increases, and the amorphous region undergoes a glass transition (Tg). The increase in molecular chain conformational entropy weakens the interaction at physical cross-linking sites, resulting in a significant decrease in the storage modulus; notably, once the temperature exceeds a certain critical value, the rubber molecular chains begin to exhibit significant entropy-elastic behavior—the molecular chains spontaneously curl under the drive of thermal motion and store elastic energy, causing the storage modulus to rise again. This phenomenon is particularly pronounced in the pineapple juice-coagulated gel, which exhibits the greatest increase in modulus in the high-temperature region. This is closely related to the sample’s broader molecular weight distribution (Figure 3) and stronger physical packing effect, as discussed earlier: at high temperatures, widely distributed molecular chains can form a more extensive entanglement network, while the low content of non-gel components reduces physical barriers between molecular chains, making the entropy-elastic contribution more prominent [32]; in contrast, traditional organic acid-cured gels form dense physical cross-linked networks due to higher protein residues, which restrict molecular chain motion; consequently, the increase in high-temperature modulus is smaller. These results confirm that the type of curing agent can significantly influence the dynamic mechanical behavior of rubber by regulating the content of non-gel components and the molecular weight distribution, particularly exerting a crucial regulatory effect on high-temperature mechanical properties.

**Figure 6:**
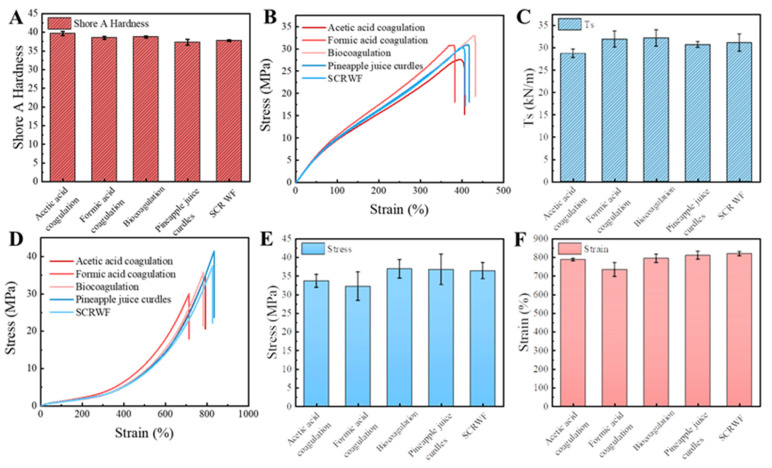
Test results for the physical and mechanical properties of vulcanized rubber: (**A**) hardness test results; (**B**,**C**) tear resistance test results; (**D**–**F**) tensile property test results.

The results of the physical and mechanical property tests shown in Figure 6 indicate that, under a uniform vulcanization formulation, natural rubber samples prepared using five different coagulants (acetic acid coagulation, formic acid coagulation, biological coagulation, pineapple juice coagulation, and SCRWF) exhibited a high degree of consistency in three key macroscopic mechanical property indicators: Shore A hardness, tensile strength, and right-angle tear strength. Specifically: the Shore A hardness values all remained stable within a narrow range of 37 ± 2 (Figure 6A), with a maximum difference not exceeding 2 hardness units; in tensile strength testing, the peak regions of the stress–strain curves showed a high degree of overlap, indicating similar tensile properties; the right-angle tear strength was concentrated in the range of 29.5–32.8 kN/m (Figure 6C), with a maximum difference of only 3.3 kN/m and a relative deviation of less than 11%. This further confirms the negligible influence of the curing process on the rubber’s tear resistance; notably, although previous dynamic mechanical analysis and vulcanization curves (Figure 5) revealed significant differences in molecular chain mobility and vulcanization kinetics among compounds prepared with different coagulants, these differences were “homogenized” by the vulcanization system in the final cured state, resulting in consistent macroscopic mechanical properties. This phenomenon indicates that the physical and mechanical properties of natural rubber are primarily determined by the vulcanized network structure, while the choice of coagulant primarily affects processing performance and microstructural characteristics, with limited impact on end-use performance under standardized vulcanization conditions. This provides natural rubber processing enterprises with an important basis for cost-performance trade-offs when selecting coagulation processes —that is, provided that the final product meets performance standards, priority may be given to the use of environmentally friendly bio-coagulants (such as pineapple juice and bio-enzymes) to reduce production costs and environmental impact. 

## 4. Conclusions

A comprehensive comparison of the overall performance among diverse solidification techniques is presented in Table 2. This study demonstrates that the coagulation method not only determines the residual state of non-rubber components in natural rubber, but also profoundly influences the aggregation behavior of its molecular chains, the formation of the gel structure, and its dynamic viscoelastic response. Compared with traditional acetic acid and formic acid coagulation systems, the pineapple juice coagulation system effectively reduces protein residues and, through the synergistic action of bioenzymes, organic acids and natural active components, regulates phospholipid content, gel network and molecular weight distribution characteristics, thereby conferring more stable green rubber properties and superior high-temperature dynamic mechanical response. However, under uniform vulcanization conditions, the hardness, tensile properties and tear resistance of vulcanized rubber obtained from different coagulation methods tend to be consistent, indicating that the vulcanization network plays a dominant role in the final mechanical properties, whilst the coagulation process primarily influences the formation of material properties by regulating microstructure and processing behavior. 

When comprehensively evaluated across four dimensions—processing stability, environmental benefits, microstructural control and final mechanical properties—traditional organic acid coagulation (acetic acid, formic acid) offers a mature process with stable final mechanical properties, but suffers from shortcomings such as high protein and phospholipid residues, weak high-temperature entropy-elastic response, high acid consumption and significant wastewater load; biological coagulants are environmentally friendly and provide good control over gel content, but have limited capacity to remove non-rubber components; the pineapple juice coagulation method, leveraging the synergistic action of bromelain and organic acids, achieves the lowest protein residue (22.5–25 mg/g), the broadest molecular weight distribution (PDI = 10.42), the most superior high-temperature dynamic mechanical response, and the best raw rubber batch stability; furthermore, under standard vulcanization conditions, its final mechanical properties are highly consistent with those of traditional acid-coagulated samples, therefore offering the most promising overall application prospects; Indonesian sheet rubber, due to its poor batch consistency, is not suitable as a stable raw material. In summary, pineapple juice coagulation and bio-coagulation technologies combine multiple advantages, including the regulation of non-rubber components, improved batch consistency of raw rubber and environmental friendliness, providing a new technical pathway for the green manufacturing of sheet rubber.

## Figures and Tables

**Table 1:** Data on molecular weight distribution.

	Formic Acid Coagulation	Acetic Acid Coagulation	Pineapple Juice Curdles	SCRWF	Bio-Coagulation	Indonesian Shag
Mn (×10^3^ g/mol)	234	200	202	251	212	238
Mw (×10^4^ g/mol)	174	169	194	156	221	163
PDI	7.42	8.47	9.61	6.21	10.42	6.85

**Table 2:** Comparison of overall performance across different solidification methods.

Parameter	Acetic Acid Coagulation	Formic Acid Coagulation	Bio-Coagulant	Pineapple Juice Coagulation	SCR WF (Control)
Coagulant type	Organic acid	Organic acid	Bio-enzyme agent	Natural plant juice	Standard coagulation process
Dosage (kg/ton dry rubber)	6–10	5–7	As per manufacturer’s recommendation	6–10	Standard process
Coagulation mechanism	pH-induced destabilization	pH-induced destabilization	Enzymatic coagulation	Protease + organic acid synergy	Acid coagulation
Protein content (mg/g)	35–37.5	37.5–40	Moderate	22.5–25	Moderate
Phospholipid content	High	High	Moderate	Relatively high	Moderate
Gel content	High	High	Moderate	Low	Moderate
Molecular weight distribution (PDI)	8.467	7.417	9.610	10.420	6.206
Batch stability of raw rubber	Fair	Fair	Good	Excellent	Good
High-temperature entropic elastic response	Weak	Weak	Moderate	Strong	Moderate
Mechanical properties of vulcanizate	Stable	Stable	Comparable to control	Comparable to control	Baseline value
Environmental benefits	High acid consumption, high wastewater load	High acid consumption, high wastewater load	Environmentally friendly	Renewable, low pollution	Conventional
Advantages	Mature process, high efficiency	Mature process, high efficiency	Environmentally friendly	Strong non-rubber component regulation, excellent batch stability, green	Stable quality
Limitations	High protein/phospholipid residue, severe wastewater pollution	High protein/phospholipid residue, severe wastewater pollution	Limited non-rubber component removal capability	Raw material affected by seasonality; enzyme activity requires standardized control	No particular advantage

## Data Availability

The original contributions presented in this study are included in the article. Further inquiries can be directed to the corresponding authors.
